# Culturable and unculturable potential heterotrophic microbiological threats to the oldest pyramids of the Memphis necropolis, Egypt

**DOI:** 10.3389/fmicb.2023.1167083

**Published:** 2023-05-18

**Authors:** Samah Mohamed Rizk, Mahmoud Magdy, Filomena De Leo, Olaf Werner, Mohamed Abdel-Salam Rashed, Rosa M. Ros, Clara Urzì

**Affiliations:** ^1^Genetics Department, Faculty of Agriculture, Ain Shams University, Cairo, Egypt; ^2^Department of Chemical, Biological, Pharmaceutical, and Environmental Sciences, University of Messina, Messina, Italy; ^3^Department of Plant Biology, Faculty of Biology, Murcia University, Murcia, Spain

**Keywords:** tangible monuments, cultural heritage, microbial genomics, microbial isolation, biodeterioration, rock-inhabiting fungi, stone-inhabiting bacteria

## Abstract

A large percentage of the world’s tangible cultural heritage is made from stone; thus, it deteriorates due to physical, chemical, and/or biological factors. The current study explored the microbial community inhabiting two prehistoric sites with high cultural value in the Memphis necropolis of Egypt (Djoser and Lahun Pyramids) using amplicon-based metabarcoding and culture-dependent isolation methods. Samples were examined by epifluorescent microscopy for biological signs before environmental DNA extraction and *in vitro* cultivation. The metabarcoding analysis identified 644 bacterial species (452 genera) using the 16S rRNA and 204 fungal species (146 genera) using ITS. In comparison with the isolation approach, an additional 28 bacterial species (13 genera) and 34 fungal species (20 genera) were identified. A total of 19 bacterial and 16 fungal species were exclusively culture-dependent, while 92 bacterial and 122 fungal species were culture-independent. The most abundant stone-inhabiting bacteria in the current study were *Blastococcus aggregatus*, *Blastococcus saxobsidens*, and *Blastococcus* sp., among others. The most abundant rock-inhabiting fungi were *Knufia karalitana* and *Pseudotaeniolina globosa*, besides abundant unknown Sporormiaceae species. Based on previous reports, microorganisms associated with biodeterioration were detected on color-altered sites at both pyramids. These microorganisms are potentially dangerous as physical and chemical deterioration factors and require proper conservation plans from a microbiological perspective.

## 1. Introduction

Tracks of human history and past civilizations are presently witnessed in archeological sites and stone monuments, which are considered an invaluable cultural heritage worldwide. A large percentage of the world’s tangible cultural heritage is made from stone. However, it is slowly but irreversibly disappearing by transforming stone into sand and soil as a part of the natural recycling process, which is essential to sustain life on Earth (Allsopp et al., 2004; Gadd, 2017). Thus, the deterioration of stone monuments represents a permanent loss of our cultural heritage.

The biodeterioration of stone is a complex process that involves biological, chemical, and environmental factors (Griffin et al., 1991; Nuhoglu et al., 2006). Although stone materials differ, their biodeterioration mechanisms are common, such as discoloration and biofilm formation (Gaylarde et al., 2012b; Martino, 2016), acid corrosion by organic and inorganic compounds (Sand and Bock, 1991; Gu et al., 2011), secondary mineral formation and crystallization redox reactions of cations (Warscheid and Braams, 2000), biological or chemical contaminants, and physical penetration by microbes (Gaylarde et al., 2006). Black crusts and dark discolorations are common symptoms of deterioration caused by microbes while biopitting, cracks, fissures, and exfoliation cause the rock’s surface to appear darker and blackish brown (Gorbushina et al., 1993; Diakumaku et al., 1995; Cappitelli et al., 2007). Melanin-producing microbes are responsible for the esthetic changes that give rocks their dark brown color (Gorbushina, 2007; Sert et al., 2007). Biodeterioration as a term that is often used to describe any damage to any stone or other objects caused by microorganisms regardless of the climatic conditions or erosion factors (Urzi, 2004; Trovão et al., 2019) with much attention given to cultural heritage stone structures (Warscheid and Braams, 2000; Gorbushina et al., 2004; Scheerer et al., 2009; Hermosin et al., 2018).

Despite the harsh conditions resulting from low water availability and nutrient concentration, stone surfaces represent a complex ecosystem of several microhabitats, enabling a diverse range of microorganisms to proliferate (Urzi et al., 2001). The microbiota reported from such complex ecosystems includes algae, fungi (including lichens), cyanobacteria, and other bacteria of various phylogenetic affiliations (Urzì et al., 2000; Lindahl et al., 2013; Piñar et al., 2019). The inhabiting microorganisms may be chasmolithic, epilithic, or endolithic (Gadd et al., 2007; Fry, 2014). Such microorganisms have been reported worldwide, including the Antarctic and regions of extreme dryness and high solar irradiation (Onofri et al., 2004); they live between the limit of adaptability and near death while barely surviving and rarely reproducing (Friedmann and Weed, 1987).

Previously, in Egyptian monuments, an environmental extremotolerant genotype of the black yeast *Hortaea werneckii* GPS5 was isolated from a stone surface in the Great Pyramid of Giza’s royal corridor (King Khufu’s pyramid; Rizk and Magdy, 2022). Three xerophilic fungi, namely *Aspergillus amstelodami*, *Aspergillus chevalieri*, and *Aspergillus repens*, and six non-xerophilic species *Alternaria alternata*, *Aspergillus terreus*, *Cladosporium herbarum*, and *Penicillium chrysogenum* were isolated from Al-Shatby and El-Anfoushi archeological tombs in the Alexandria governorate (Alexandria city), respectively (Afifi and Geweely, 2011). Fungal damage (e.g., decayed wood, contaminated bones, and black spots on mud objects) was observed in Tuna El-Gabel’s excavations near Al-Minya city, where different organic and inorganic materials belonging to the Ptolemaic era were found (Mansour and Ahmed, 2012). The identified fungal isolates were *A*, *alternata*, *Aspergillus flavus*, *Aspergillus niger*, *Bipolaris sorokiniana*, *Dichotomopilus indicus*, *Fusarium fujikuroi*, and *Rhizopus ehrenb*.

*Aspergillus niger* and *A*, *terreus* were the most common and dominant fungal deteriogens, followed by *Aspergillus fumigatus*, *Cladosporium cladosporioides*, and *C*, *herbarum* in Seti Ι tomb (Abydos city), Senusret Ι obelisk (Fayoum city), Great pyramid complex (Giza city), Mosque of judge Abd El-Basset (Cairo city), and Museum of Ismailia Antiquities (Ismailia City) where fungal biodeterioration signs were uncovered (e.g., black spots; Mohamed and Eid Ibrahim, 2018). Brown spots on the famous Tutankhamun’s tomb walls were investigated for possible microbial origin (Vasanthakumar et al., 2013). Those authors found that fungal communities were composed primarily of Genera *Penicillium*, whereas the abundant bacterial taxa were members of the Firmicutes, Actinobacteria, and Bacteroidetes phyla. Actinobacteria demonstrated a great taxonomic diversity on stone surfaces. Despite the predominance of isolates of the genus *Streptomyces*, members of the genera *Geodermatophilus* and *Rhodococcus* were also reported (Groth et al., 1999). In addition to *Streptomyces* and *Nocardia*, *Micromonospora* species were also isolated from ancient stones from a tomb site in Tell Basta (Zagazig city, Egypt (Abdulla et al., 2008)).

Previously, culture-dependent (traditional isolation methods) were used to identify the organisms associated with the discoloration and degradation of historic buildings. By using this method, inactive forms and unculturable species are not considered, while active conditions are detected; thus, only a small fraction of the total diversity of microbes can be detected (Urzì et al., 2000, 2010; Urzi, 2004; de Los Ríos and Ascaso, 2005). Microbial biodiversity in sandstone surfaces was further investigated using next-generation sequencing (NGS) for profiling microbial populations in biodeterioration cultural heritage studies, which can provide a solution to the limitations imposed by the culture-dependent method (Gaylarde et al., 2012a; Ogawa et al., 2017; Soliman and Magdy, 2018; Trovão et al., 2019; Skipper et al., 2022).

The current study’s general objective was to reveal part of the microbial diversity inhabiting two prehistoric sites in the Memphis necropolis of Egypt (Djoser and Lahun Pyramids) with high cultural values exposed to harsh and arid environmental conditions. The specific aims were the following: (i) to explore the nature, richness, and diversity of the bacterial and fungal microbiota inhabiting Djoser and Lahun Pyramids, (ii) to check if extremotolerant species with potential biodeterioration effects are present, and (iii) to compare the effectiveness of the amplicon-based metabarcoding analysis and traditional isolation methods to detect biodeterioration-associated microbes of the studied pyramids.

## 2. Materials and methods

### 2.1. Archeological sites

Geographically, the sampling area is generally characterized by light, warm, dry sandy soil that tends to be acidic with low nutrients (Mahmoud et al., 2015) and with an arid climate characterized by high UV exposure and day temperature that drop drastically during the night (The Egyptian Meteorological Authority, http://ema.gov.eg/wp/). Two pyramids were chosen for this study; they are among the oldest and largest ones in the Memphis necropolis of ancient Egypt. Deterioration was observed in many parts of both pyramidal complexes in the form of dark spots, coloration, and brittle rocks.

The pyramid of Djoser (DP), also known as the “Step Pyramid,” is an archeological remain in the Saqqara district in Memphis necropolis, located in the northern part of the Nile Valley, northwest of the city of Memphis, situated at 29°52′10.17”N and 31°13′8.70″E in Giza governorate, Egypt The building was constructed during the 27th century B.C. (3rd dynasty) of limestone by Imhotep, King Djoser’s vizier (Mark, 2016). In an enormous courtyard surrounded by ceremonial structures and decorations, it is the focal point of a vast mortuary complex (Shaw, 2003). It is considered the first Egyptian pyramid and is known as the world’s oldest structure, built entirely of stone (Hawkes, 1974). The Pyramid of Lahun (commonly spelled Al-Lāhūn; LP), also known as the “Senusret II Pyramid” or the “Mud-pyramid,” is located on the west bank of the Nile valley near the opening of the Hawara Channel from the Nile Valley into the Fayum basin situated at 29°14′0″N and 30°58′0″E. The construction of the pyramid is believed to have been constructed by the pharaoh Senusret II ~ 2,000 BC (12^th^ dynasty) and is considered the first large mudbrick Pyramid with a yellow limestone core. It was once covered entirely by white limestone (Redford, 2005). The wall had been encased in limestone that was decorated with niches, perhaps as a copy of Djoser’s complex at Saqqara; although it is still impressively large, A natural outcrop of the pyramid can be seen now in its ruinous condition of the yellow limestone core can be seen protruding from the rubble of the mudbrick fill in some places (Eugene Cruz-Uribe, 2010).

### 2.2. Sampling

**Figure 1:**
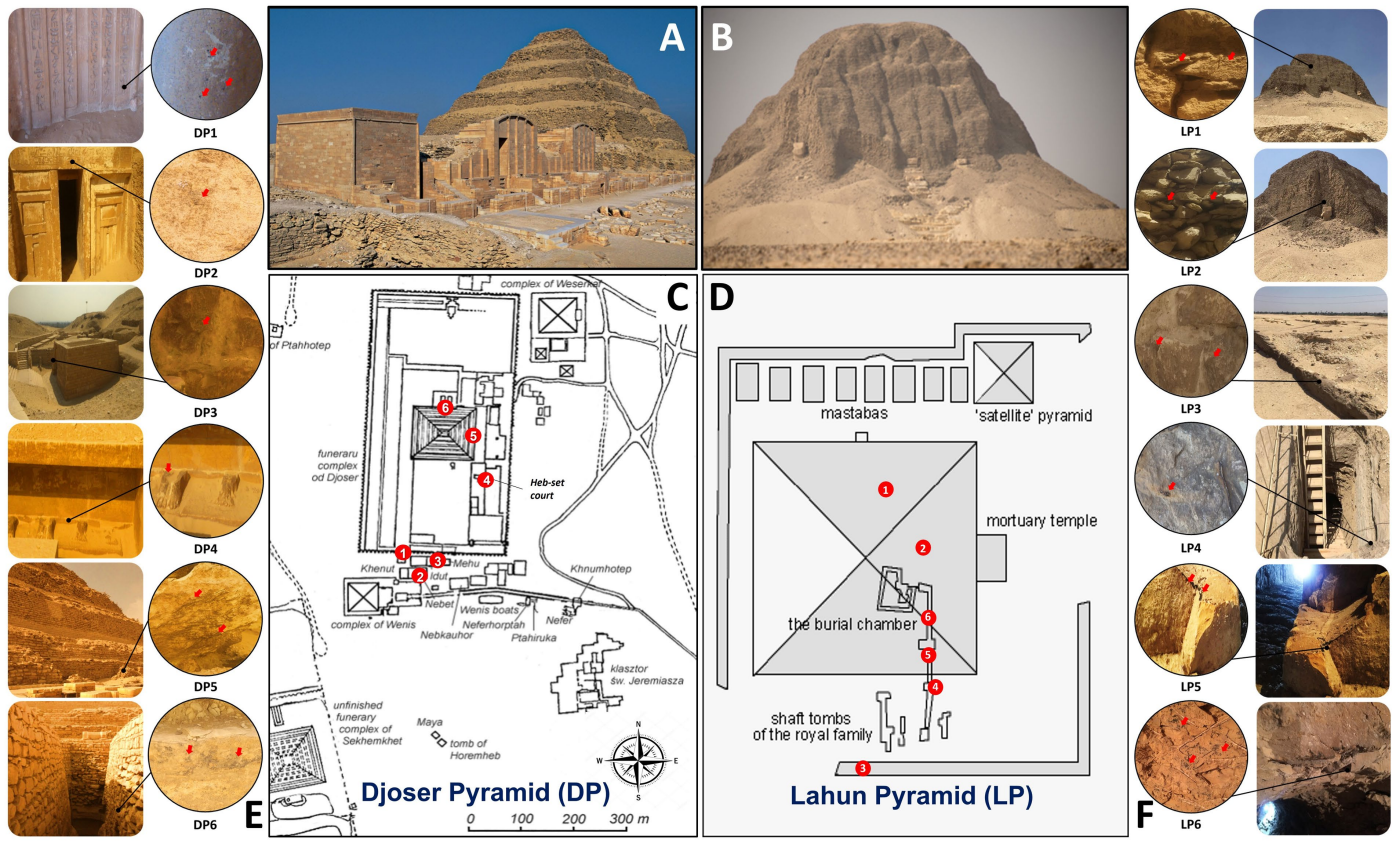
Sampling site information in Djoser pyramid (DP) and Lahun pyramid (LP). **(A,B)**: photographs of DP and LP, respectively, (by Samah Mohamed Rizk). **(C,D)**: schematic drawing of DP and LP complexes, respectively, showing the sampling sites by numbers (Table 1). **(E,F)**: sampling sites photographs showing their location in the pyramid and the sampling spot (by Samah Mohamed Rizk).

Six samples per pyramid were collected around the archeological sites (Table 1; Figure 1); no special permission was required, as the sampling area is a free-walking zone, and the sampling was performed using a non-destructive method. Samples were collected using a sterilized scalpel and brush. Soft scratches of the brittle rock formations and sandy soil from the stone structures’ that showed different biodeterioration types, such as biopitting, and black crusts or spots in samples (DP1, DP2, DP5 and DP6; LP3, LP4, LP5 and LP6). Erosion and mild color alteration, mostly gray to brown, as observed in samples (DP4, LP1 and LP2). The samples were placed into 50 mL Falcon tubes and preserved in sterile bags for further analysis. A portion of ~1 g was aliquoted from each sample and preserved in −20°C for the amplicon-based metabarcoding analysis.

**Table 1:** Nature and location description of the collected samples in Djoser (DP) and Lahun (LP) pyramids; sample codes are the same as in Figure 1.

Code	Nature and location of samples in pyramids
DP1	Scratch from the entrance tablet of an unidentified tomb near Mastaba “Khenut”
DP2	Scratch from the false door of the Mastaba “Khenut” southwest corner
DP3	Sand covers from Mastaba “Mehu” entrance
DP4	Sand covers the feet of four statues found in the “Heb-Sed” court
DP5	Brittle rock precipitations of the western Massifs on the pyramid’s left side
DP6	Sand cover from the entrance ground
LP1	Sandy soil on the surface of the mud-rocks from the top of the pyramid
LP2	Sandy soil on the surface of the rocks from the south-eastern limestone stump
LP3	Soil from the queen’s pyramid remains
LP4	Scratch of altered rock surface at the entrance shaft
LP5	Scratch of altered rock surface corridor to the burial chamber
LP6	Scratch of altered rock surface at the ceiling of the ventilation room

### 2.3. Sample preparation and epifluorescence examination

Each sample was powdered in a mortar, and ~1 g was suspended (1,10) in a physiological solution (i.e., isotonic solution: 0.9% NaCl) with the addition of 0.001% Tween 80 and continuously agitated for 1 h at 30°C to facilitate a better separation and distribution of microorganisms living in/on the rock material (Urzi et al., 2001). Epifluorescent microscope examination was performed using a drop of sample suspension prepared at the previous step and a drop of 0.1% (w/v) of Acridine Orange solution. Direct observations of samples were carried out using a light/epifluorescent Leica DMR microscope equipped with a 50 W mercury lamp and a 450–490 nm excitation filter.

### 2.4. DNA extraction and metabarcoding analysis

Total environmental DNA (eDNA) was extracted directly from 0.25 g of each sample using the PowerSoil DNA Isolation Kit (MO BIO Laboratories Inc., CA, USA) and quantified using a Qubit fluorometer and the Qubit BR assay kit (Invitrogen, Life Technologies, USA). Due to the low concentration of the obtained eDNA from the current Material, for each pyramid, samples were bulked into two sets (including three field-collected samples each) to reach the minimum concentration level required for the metabarcoding library construction (Supplementary Tables S1, S2).

For the bacteria, the V3-V4 region rRNA of the bacterial 16S RNA gene was amplified using primers 338F (5′-ACT CCT ACG GG AGG CAG CAG-3′) and 806R (5′-GGA CTA CHV GGG TWT CTA AT-3′; Caporaso et al., 2010). For the fungi, the ITS1 of the nuclear ribosomal RNA genes was amplified using primers ITS1F (5’-TCC GTA GGT GAA CCT GCG G-3′) and ITS2R (5’-GCT GCG TTC TTC ATC GAT GC-3′) as recommended for the Miseq analysis (Blaalid et al., 2013). Four independent PCR assays were performed for each bulked DNA sample under the following conditions: a 20 μL PCR reaction using TransStart FastPfu DNA Polymerase mixture contained 4 μL of 5× FastPfu Buffer, 2 μL of 2.5 mM (each) dNTPs, 0.8 μL of 5 μM Bar-PCR primer F, 0.8 μL of 5 μM primer R, 0.4 μL of FastPfu polymerase, 0.2 μL of BSA and 10 ng of genomic DNA. PCR amplification was conducted in an ABI GeneAmp 9,700 thermocycler (IET, USA) under the following conditions: 98°C for 3 min, 27 cycles of 10 s at 98°C, 60°C for 30 s, and 72°C for 45 s, followed by 7 min at 72°C. PCR products were examined by 2% agarose gel electrophoresis and purified using Agencourt AMPure XP beads (Beckman, USA) and were sequenced by Illumina (MiSeq, PE 2 × 300 bp mode), following Illumina instructions. Using FLASH (Magoc and Salzberg, 2011) and Trimmomatic (Bolger et al., 2014), the pair-end reads were trimmed at any sites receiving an average quality score below 20 over a 50 bp sliding window, while reads shorter than 50 bp were discarded. The pair ends were merged with a minimum overlap length of 10 (0.2 maximum mismatch ratio). Barcodes and primer sequences at both ends were used to obtain valid sequences per sample, with 0 and 2 allowed mismatches, respectively.

The metabarcoding analysis was performed using the online Majorbio Cloud Platform.^1^ The operational taxonomic units (OTUs) were retrieved as follows: sequences with a single occurrence were discarded, and the remaining sequences were dereplicated and tested for chimeric sequences and artifacts using the UPARSE software (version 7.1 http://drive5.com/uparse/) at OTU sequence similarity of 0.97. The retained representative sequences were classified using the RDP Classifier^2^ and mapped against the Silva (Release 138 http://www.arb-silva.de) database for 16S rRNA and Unite (Release 8.3 http://unite.ut.ee/index.php) for ITS using a confidence threshold of 0.7. Microbial composition statistics were estimated at each taxonomic level (domain, kingdom, phylum, class, order, family, genus, and species). Mothur (Schloss et al., 2009) was used to assess species richness and microbial diversity, while the Venn diagrams and bar plots for the microbial composition were analyzed and visualized using the vegan R package.^3^ The species percentage per sample shown as a circos plot was elaborated using Circos-0.67.^4^ The OTU abundance table was analyzed by PICRUSt and FUNGuild to predict the microbial function annotations. Heatmaps were constructed using Orange 3.24.1.^5^

### 2.5. Culture-dependent isolation of microorganisms

#### 2.5.1. Isolation and enumeration

For the isolation and the enumeration of cultivable chemoorganotrophic microorganisms in the 12 field-collected samples, the pour plate technique (Burdass et al., 2006) was applied by inoculating 1 mL of the sample suspension (prepared in the sample preparation step) following decimal dilutions 10P^−1^P:10P^5^P. The sample suspension was poured in duplicates onto (1) BRII medium (Bunt and Rovira, 1955) modified as reported by Urzi et al. (2001) + 0.05% of the antifungal cycloheximide was utilized for chemoorganotrophic bacteria (Atlas, 2005); and (2) Geo medium (#714, List of Media for Microorganisms, DSMZ, Germany) + 0.05% cycloheximide was utilized for *Geodermatophilus* species (Atlas, 2005). The plates were then incubated at 28°C for up to 1 month. For the isolation and the enumeration of cultivable fungi, DRBC medium (Dichloran Rose Bengal Chloramphenicol agar; #CM0727, Oxoid, USA) supplemented with 100 ppm chloramphenicol to prevent bacterial growth (#SR0078, Oxoid, USA) was used. Incubation was carried out at 25°C for up to 1 month. At the end of incubation time, counts of viable microorganisms were referred to as colony-forming units *per* gram of sample (CFU/g) to detect the microbial community’s enumeration in samples per each g for bacteria and fungi (Urzì et al., 2010).

#### 2.5.2. Molecular identification of microbial isolates

DNA extraction from successfully isolated strains was performed using the PureLink Genomic DNA kit according to the kit protocol. DNA quality was checked using 1% (w/v) agarose gel electrophoresis, visualized by pre-added RedSafe dye under UV light, and quantified using Qubit and the Qubit BR assay kit (Invitrogen, Life Technologies, USA).

For the isolated bacteria, the bacterial 16S rRNA gene was fully amplified using primers 27F (5′-AGA GTT TGA TCC TGG CTC AG-3′) and 1,492R [5′-GGT TAC CTT GTT ACG ACT T-3′ (Lillo et al., 2006)]. For the isolated fungi, the ITS1 of the nuclear ribosomal RNA genes was amplified using primers ITS1F (5′-TCC GTA GGT GAA CCT GCG G-3′) and ITS4R [5′-TCC TCC GCT TAT TGA TAT GC-3′ (White et al., 1990)]. PCR reactions were performed using the Red Mix (BioLine, UK) kit. Each 25 μL reaction tube included 5 pmol of each primer, and 40 ng of DNA template was added. The amplification was carried out using a Techne 512 thermocycler (Techne, UK). The PCR program was adjusted according to the primer pair melting temperature (Tm) as follows: the first denaturation step at 95°C for 3 min was followed by denaturation at 95°C for 2 s, annealing at 50°C for 30 s for 16S rRNA and at 55°C for 30 s for ITS, extension at 72°C for 30 s. The last three steps were repeated 35 times, with the last extension step of 72°C for 5 min. PCR products were tested using 1.5% agarose gel electrophoresis and prepared for purification using GeneJET PCR purification kit (K0702, Fermentas, USA) before automated Sanger sequencing.

Chromatograms were trimmed, assembled, and aligned using Geneious Prime (Kearse et al., 2012) and blasted for species identification using NCBI online Blast tool^6^ against the ITS database using the default settings. Taxonomic ranking and phylogenetic relationships were retrieved from the taxonomy database.^7^

## 3. Results

### 3.1. Epifluorescence examination

With the aim to explore the biological presence in the samples collected from such hyper-arid locations, the samples were examined by direct epifluorescent microscopy. Evidence of bacterial cells and fungal spores among the particles of the soil collected from both pyramids was found. The examination showed a uniform green stain in the case of bacteria, whereas phototrophic cytoplasm showed a red autofluorescence due to the presence of chlorophylls; the nuclei of eukaryotic cells appeared green, while the cytoplasm of heterotrophs was orange (Supplementary Figure 1).

### 3.2. Environmental DNA extraction

The eDNA was extracted successfully for the 12 field-collected samples. The eDNA concentration ranged from 19 to 49 ng/μl with an average of 36.85 ± 10.97 ng/μl for the DP samples and from 26 to 60 ng/μl with an average of 40.33 ± 10.96 ng/μl for the LP samples.

### 3.3. Metabarcoding analysis of the bacterial 16S rRNA

#### 3.3.1. Raw reads information

After filtering, the four bulked samples (two sets per pyramid) recorded an average of 43,881 ± 3,318 sequence reads, with an average nucleotide number of 18 ± 1.5 million nucleotides. The mean read length ranged from 413 to 422 bp; the minimum recorded sequence read length was 226 (bulk LP_S1), while the maximum was 526 bp (bulk LP_S2; Supplementary Table 1). The total number of sequences was 232,423, with an average length of 417 bp. Using an alignment threshold of 97% similarity level, the number of classified sequences was 225,266 (96.92%), while only 7,157 sequences had ‘no known relative’ (3.08%).

#### 3.3.2. Taxonomical composition

Based on the OTU identification pipeline and the Venn diagram plot, the total number of the identified OTUs in the metabarcoding samples (meta) was 940. Both pyramids shared 284 OTUs, while 104 and 552 were uniquely found in DP and LP, respectively. The OTUs identified from the two pyramids DP and LP were 388 and 836, respectively (Supplementary Figure 2).

**Figure 2:**
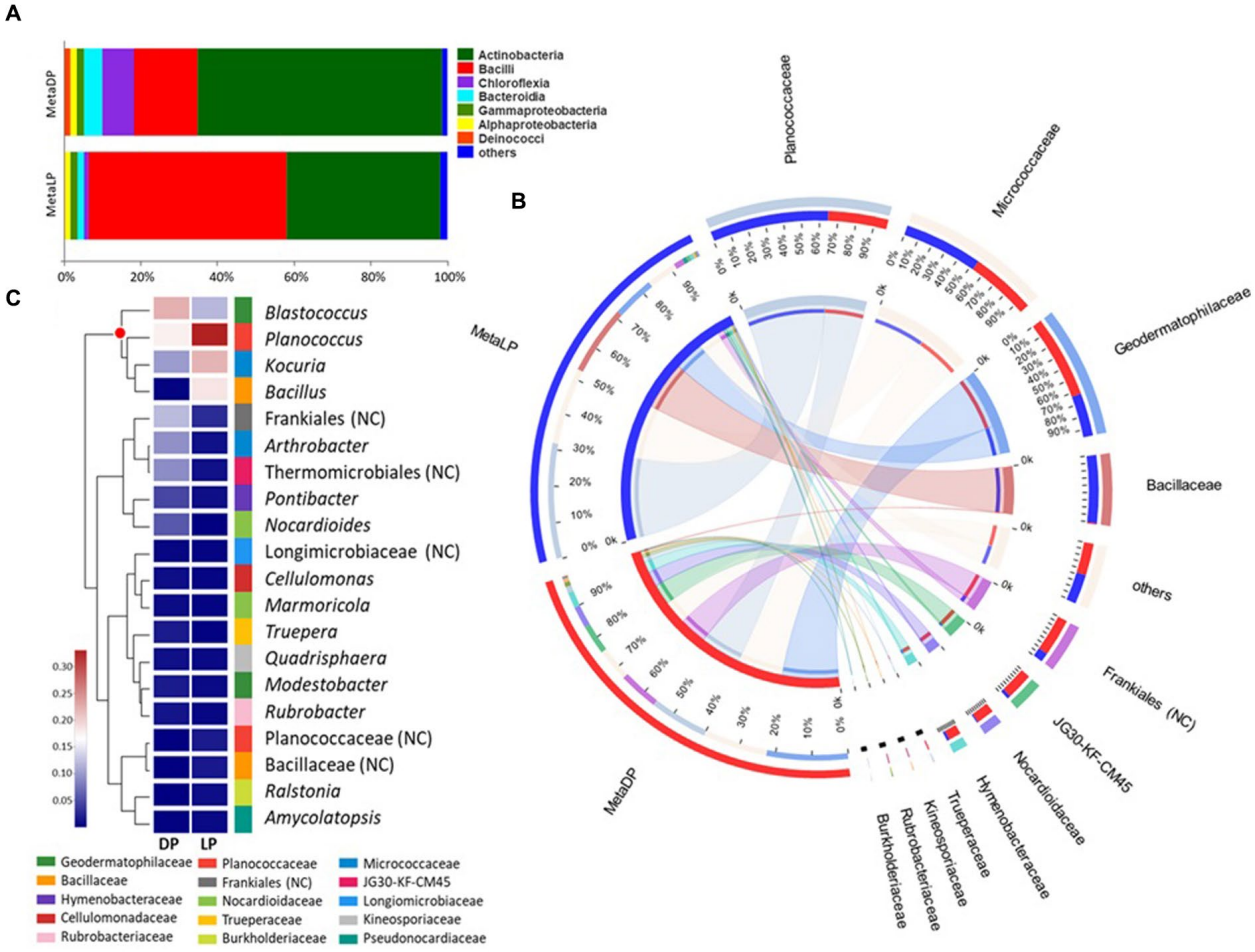
16S rRNA-based metabarcoding profiling of Djoser and Lahun pyramids. **(A)** Bacterial community composition bar-plot based on the identified OTUs for DP and LP in the metabarcoding samples. The percentage of community abundance at the class level is shown. **(B)** Circos plot for the comparative bacterial community composition based on the identified OTUs for both DP (MetaDP; red) and LP (MetaLP; blue) in the metabarcoding samples. The percentage of community abundance at the family level is shown for each pyramid. NC = not classified. **(C)** Bacterial community heatmap for the top 20 of the most abundant genera. The relative abundance ratio is shown for the two pyramids (DP and LP) using a colour scale given on the left of the figure. The family is indicated by colour mark for each genus. The top represented genera are clustered and marked by the red dot. NC = not classified.

The identified OTUs were classified into higher taxonomical ranks. Six hundred and forty four out of the 940 OTUs were classified as species belonging to 452 genera, forming 256 families from 142 orders, 57 classes, and 26 phyla, and all belonged to kingdom Bacteria. The relative proportion of the major bacterial classes was visualized by abundance for each pyramid. For both pyramids, the Actinobacteria and Bacilli were the most abundant classes; however, the percentage values’ order was reversed between both pyramids. In the case of DP, the class Actinobacteria was the most abundant, followed by the Bacilli, Chloroflexia, Bacteroidia, Gammaproteobacteria, Alphaproteobacteria, and Deinococci classes, among others. On the contrary, in LP, the class Bacilli was the most abundant, followed by Actinobacteria, Bacteroidia, Gammaproteobacteria, Alphaproteobacteria, and Chloroflexia classes, among others (Figure 2A).

When the community composition was compared between both pyramids at the family level, OTUs belonging to three major families were highly represented in both communities (i.e., at least >1% abundance in both pyramids), namely: Geodermatophilaceae, Micrococcaceae, and Planococcaceae. Additionally, the OTUs belonging to the family Bacillaceae were highly represented in LP while weakly found (<1%) in DP. In contrast, OTUs belonging to orders Frankiales (non-ranked) and JG30-KF-CM45 (Thermomicrobiales), and families Hymenobacteraceae and Nocardioidaceae, among others, were highly represented in DP while much rarer (<1%) in LP (Figure 2B). By testing the taxonomical relationships among the detected families, five bacterial classes were highly represented. Class Actinobacteria was the most represented (five families), followed by Bacilli (two families), Bacteroidia, Chloroflexia, and Gammaproteobacteria (single family).

Based on the metabarcoding analysis, the top-represented bacterial genera were *Blastococcus*, *Planococcus*, *Kocuria*, and *Bacillus*, respectively. According to the applied database classification, the genus *Blastococcus*, family Geodermatophilaceae, was represented by *Blastococcus saxobsidens*, with a relative abundance ratio (rA) of 0.155 (DP) and 0.104 (LP), *Blastococcus aggregatus* with an rA of 0.054 (DP) and 0.011 (LP), and an unclassified *Blastococcus* sp. with an rA of 0.034 (DP) and 0.011 (LP). The genus *Planococcus*, family Planococcaceae was represented by *Planococcus salinarum* with an rA of 0.167 (DP) and 0.126 (LP) and an unclassified *Planococcus* sp. with an rA of 0.024 (DP) and 0.224 (LP). *Kocuria rosea* represented the genus *Kocuria*, family Micrococcaceae with an rA of 0.109 and 0.234 for the DP and the LP samples, respectively. The genus *Bacillus*, family Bacillaceae was represented by *Bacillus persicus* with an rA of 0.001 (DP) and 0.129 (LP) and *Bacillus alkalitelluris*, with an rA of 0.008 × 10P^−1^P (DP) and 0.050 (LP; Figure 2C).

The overall functional composition profile was predicted for each pyramid based on the identified OTUs. Both profiles were very similar; the relative abundance was the highest for bacteria characterized by genes of unknown function, general function and amino acid transport and metabolism, energy production, and conversion, among others. In the latter, minor differences were found between both pyramids, as DP was slightly higher than LP (Supplementary Figure 3).

### 3.4. Metabarcoding analysis of the fungal its region

#### 3.4.1. Raw reads information

After filtering, the four bulked samples recorded an average of 49,483 ± 7,111 sequence reads, with an average nucleotide number of 12.47 ± 1.67 million nt. The mean read length ranged from 251 to 254 bp; the minimum recorded sequence read length was 140 (bulk LP_S2), while the maximum was 515 bp (bulk DP_S1; Supplementary Table 2). Total valid sequences were 98.5 K and 133.5 K for the DP and the LP, respectively. Using an alignment threshold of 97% similarity level, the number of classified sequences was 301,603 (99.52%), while only 1,468 sequences had ‘no known relative’ (0.48%).

#### 3.4.2. Taxonomical composition

**Figure 3:**
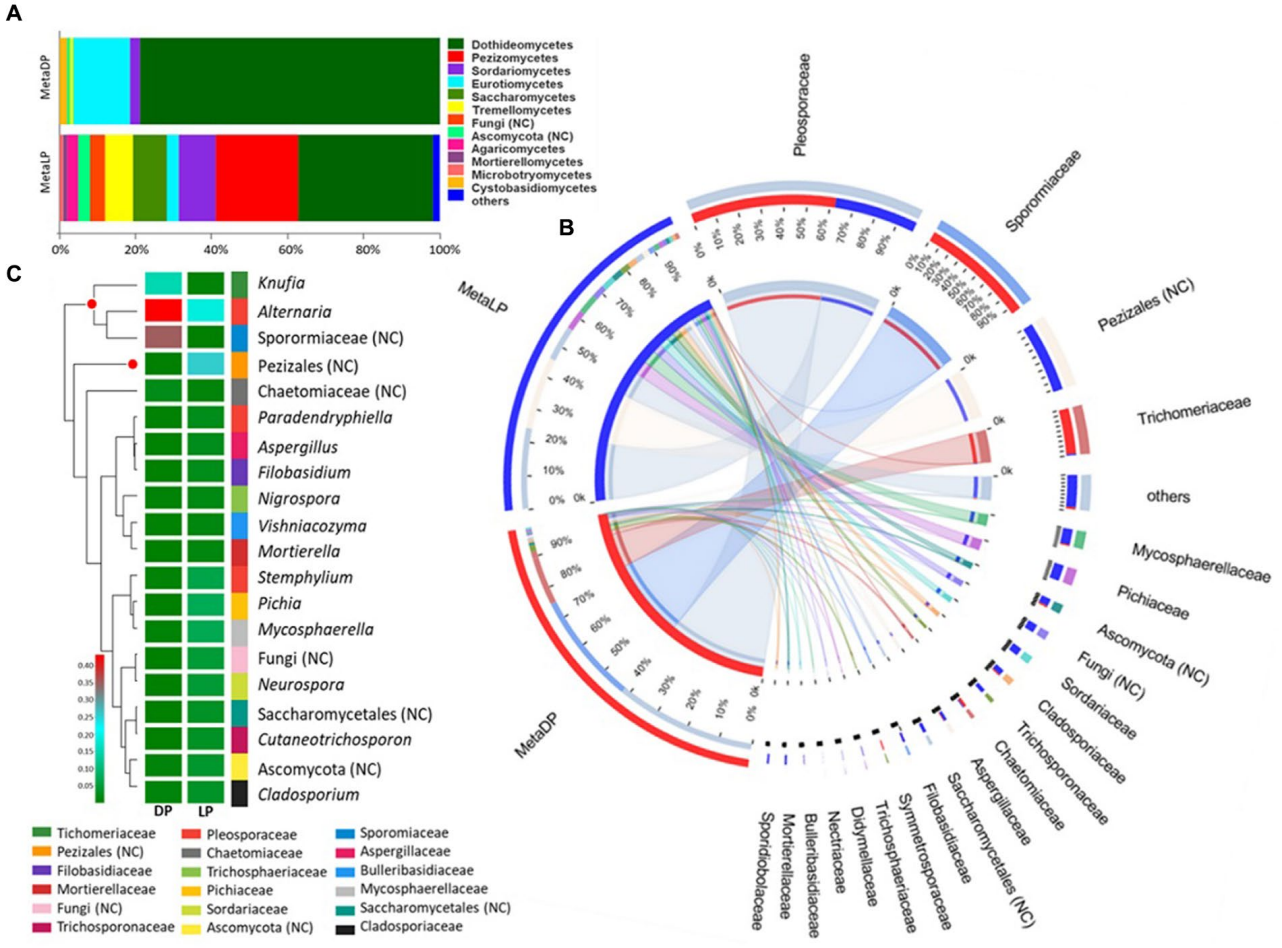
ITS-based metabarcoding profiling of Djoser and Lahun pyramids. **(A)** Fungal community composition bar-plot based on the identified OTUs for DP and LP in the metabarcoding samples. The percentage of community abundance on the class level is shown. **(B)** Circos plot for the comparative fungal community composition based on the identified OTUs for both DP (MetaDP; red) and LP (MetaLP; blue) in the metabarcoding samples. The percentage of community abundance at the family level is shown for each pyramid. NC = not classified. **(C)** Fungal community heatmap for the top 20 of the most abundant genera. The relative abundance ratio is shown for the two pyramids (DP and LP) using a color scale given on the left of the figure. The family is indicated by color for each genus. The top represented genera are clustered and marked by a red dot. NC = not classified.

Based on the OTU identification pipeline and the Venn diagram plot, the total number of the identified OTUs in the metabarcoding samples was 306. Both pyramids shared 48 OTUs, while 43 and 215 OTUs were uniquely found in DP and LP, respectively. The OTUs identified from the two pyramids DP and LP were 91 and 263, respectively (Supplementary Figure 4). The identified OTUs were classified into higher taxonomical ranks; 204 out of the 306 OTUs were classified as species belonging to 146 genera, forming 99 families, 48 orders, 21 classes, and 6 phyla, all belonging to the kingdom Fungi. The relative proportion of the major fungal classes was visualized by abundance for each pyramid. For both pyramids, the class Dothideomycetes was the most abundant. However, in DP, it was up to 80% compared to LP with 37%. In the case of DP, this class was followed by the Eurotiomycetes and Sordariomycetes classes, among others. In LP, the Dothideomycetes were followed by the Pezizomycetes, Sordariomycetes, Saccharomycetes, Tremellomycetes, and Eurotiomycetes classes, among others (Figure 3A).

When the community composition was compared between both pyramids at the family level, one family was highly represented in both communities (i.e., at least >1% abundance in both pyramids), namely: Pleosporaceae. Additionally, the families Sporormiaceae and Trichomeriaceae were highly represented in DP while weakly found (<1%) in LP. In contrast, the families Cladosporiaceae, Mycosphaerellaceae, Pichiaceae, Sordariaceae, and Trichosporonaceae, among others, were highly represented in LP while weakly found (<1%) in DP (Figure 3B). Besides, OTUs classified to order Pezizales and others belonging to phylum Ascomycota were highly represented in LP while weakly found (<1%) in LP. The abundant families represented five classified fungal classes and two unclassified ones by testing the phylogenetic relationships among the detected families. The class Dothideomycetes was the most represented (four families), followed by Eurotiomycetes (two families), Pezizomycetes, Saccharomycetes, and Sordariomycetes (single-family).

In descending order, the top represented fungal genera based on the metabarcoding analysis were: *Knufia*, *Alternaria* an unclassified member of the Sporormiaceae family (GenBank accession: MN899880), and an unclassified member of Pezizales order (UNITE DOI:SH1569317.08FU). The genus *Knufia* was represented by *Knufia karalitana*, family Trichomeriaceae with an rA of 0.151 and 0.005 for the DP and the LP, respectively. *Alternaria chlamydospora* with an rA of 0.122 (DP) and 0.007 (LP) and *Alternaria oudemansii* with an rA of 0.301 (DP) and 0.009 (LP) represented the genus *Alternaria*, family Pleosporaceae. The unclassified species of the family Sporormiaceae showed rA values of 0.35 for the DP and 0.001 for the LP samples. The Pezizales unclassified species showed rA values of 0.007 × 10P^−2^P for the DP and 0.271 for the LP samples (Figure 3C).

The overall functional composition profile was predicted for each pyramid based on the identified OTUs. Both profiles were very different; the relative abundance was the highest for fungi characterized as an animal pathogen, dung saprotrophs, and unknown or undefined saprotroph types. For the DP, the fungi characterized as animal pathogens or dung saprotrophs were higher represented than for the LP, similar to those belonging to the “Animal-Pathogen—Endophytic—Lichen Parasite—Plant-Pathogen—Wood saprotroph” group. In contrast, the fungi of unknown or undefined saprotroph type and those characterized as plant pathogens were more prevalent in LP than DP samples (Supplementary Figure 5).

### 3.5. *In vitro* culture analysis and traditional isolation of microorganisms

#### 3.5.1. Enumeration of most representative colonies

Variable numbers of cultivable bacteria and fungi among the DP samples were observed. The CFU/g ranged between 1.63 and 2.33 for bacteria and 1.78 to 2.41 for fungi (DP1 was excluded since no cultivable fungi were found). The highest number of bacterial and fungal isolates was observed in sample DP6, while the lowest was found in sample DP1. Total cultivable bacteria in DP samples were 61 on BRII and 144 on Geo media, with total cultivable fungal colonies of 211. The CFU/g ranged between 1.82 and 2.22 for bacteria and 2.00 and 2.48 for fungi compared to LP samples. The highest number of bacterial and fungal isolates was observed in sample LP1, while the lowest was found in sample LP2. Total cultivable bacteria in the LP samples were 96 on BRII and 106 on Geo media, with total cultivable fungal colonies of 278. The LP samples showed a higher number of successfully identified bacteria 32 than the DP of 20 and fungi of 40 for the LP to 26 for the DP samples (Table 2).

**Table 2:** Enumeration of the cultivable colonies isolated from Djoser (DP) and Lahun (LP) pyramids.

Samples	Bacteria (CFU/g)	Fungi (CFU/g)	Isolate counts						
			a	b	c	d	e	f	g
DP1	2.22	0.00	-	33	0	-	-	3	-
DP2	2.33	1.78	24	17	12	-	1	5	2
DP3	2.30	2.41	-	40	45	-	-	4	3
DP4	2.23	2.18	-	34	30	-	-	3	3
DP5	1.63	2.23	13	-	51	1	1	3	8
DP6	2.12	2.26	24	20	73	-	-	2	10
Total DP	**-**	**-**	61	144	211	1	2	20	26
LP1	2.12	2.48	40	-	90	2	4	5	8
LP2	1.83	2.10	26	-	38	-	2	5	7
LP3	2.22	2.00	-	50	30	1	-	6	5
LP4	2.00	2.06	30	-	35	-	-	6	5
LP5	1.82	2.18	-	20	45	-	2	4	10
LP6	2.08	2.12	-	36	40	-	4	6	5
Total LP	**-**	**-**	96	106	278	3	12	32	40

(a) Total cultivable bacterial colonies on BRII medium. (b) Total cultivable bacterial colonies on Geo medium. (c) Total cultivable fungal colonies on DRBC medium. (d) Count of occasionally found bacteria. (e) Count of occasionally found fungi. (f) Count of successfully identified bacteria using 16S. (g) Count of successfully identified fungi using ITS.

#### 3.5.2. Molecular identification of microbial isolates

The isolation approach surveyed 28 bacterial species (13 genera) and 34 fungal species (20 genera). In the case of Djoser Pyramid, the bacterial 16S rRNA identified 13 strains among the successful isolates, and the fungal ITS region rRNA enabled the identification of 15 isolates that were amplified and analyzed. At the genus level, the most frequent bacteria belonged to 8 genera, namely: *Arthrobacter*, *Bacillus*, *Brevibacterium*, *Kocuria*, *Micrococcus*, *Pseudomonas*, *Streptomyces*, and *Xanthomonas* (Supplementary Table 3). Equally, the isolated fungi were assigned to 10 genera, namely: *Alternaria*, *Aspergillus*, *Cladosporium*, *Curvularia*, *Epicoccum*, *Fusarium*, *Glomerella*, *Penicillium*, *Phialocephala*, and *Ulocladium* (synonym of *Alternaria*; Supplementary Table S4). A black meristematic fungus was isolated from samples DP5 and DP6 and was assigned to the genus *Pseudotaeniolina*.

In the case of Lahun Pyramid, the bacterial 16S rRNA identified 21 strains among the successful isolates, and the fungal ITS region enabled the identification of 26 amplified and analyzed strains. At the genus level, the most frequent strains of bacteria belonged to 13 genera, namely: *Agrobacterium*, *Arthrobacter*, *Bacillus*, *Brevibacterium*, *Clostridium*, *Klebsiella*, *Kocuria*, *Micrococcus*, *Micromonospora*, *Pseudomonas*, *Rhizobium*, *Streptomyces*, and *Xanthomonas* (Supplementary Table 4). The identified fungi were assigned to 16 genera: *Alternaria*, *Aspergillus*, *Chaetomium*, *Cladosporium*, *Curvularia*, *Epicoccum*, *Fusarium*, *Monilinia*, *Mucor*, *Mycosphaerella*, *Podospora*, *Puccinia*, *Stachybotrys*, *Stemphylium*, *Trichoderma*, and *Ulocladium* (Supplementary Table 4).

#### 3.5.3. Comparative phylogenetic and taxonomic analyses

**Figure 4:**
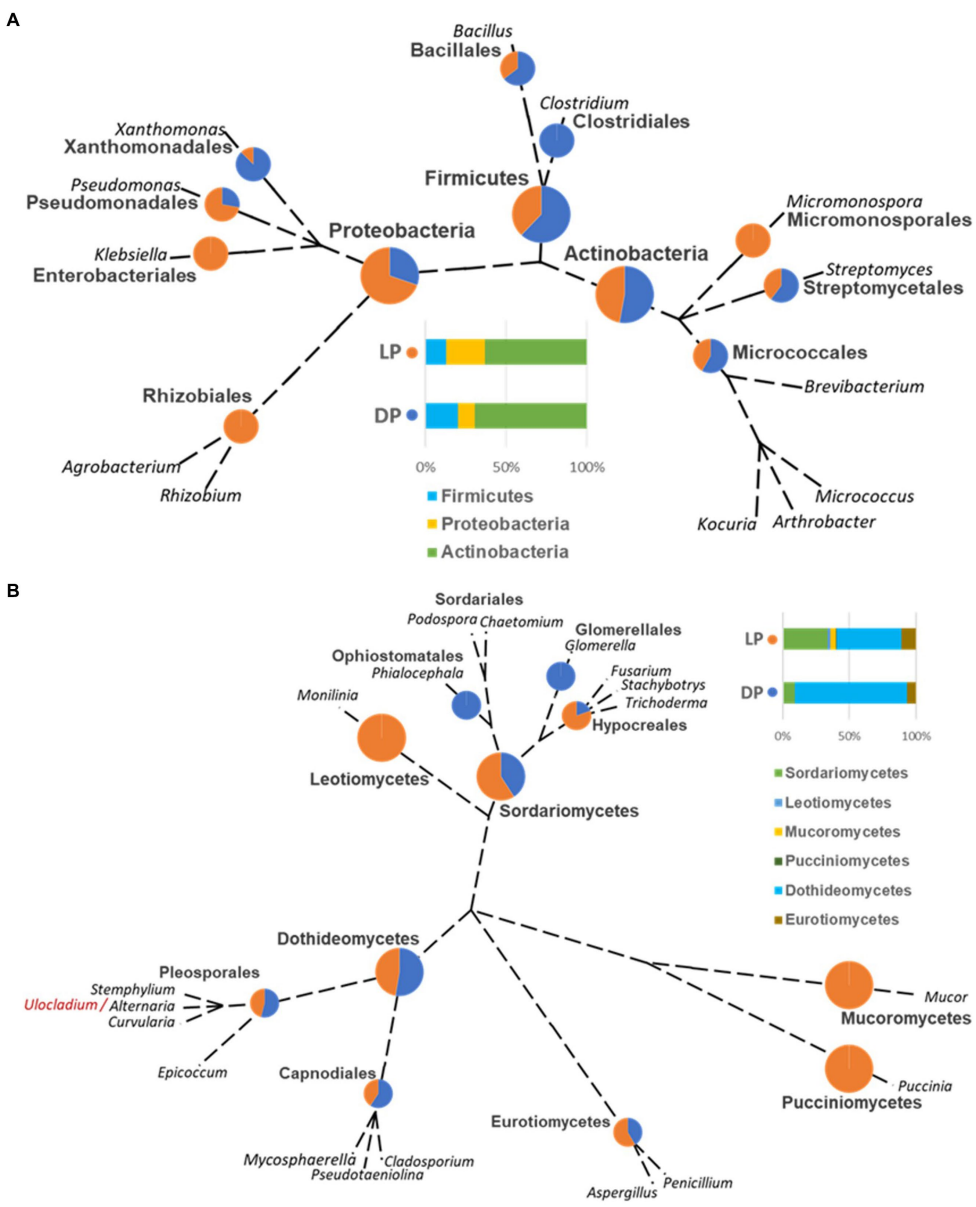
Unrooted phylogenetic tree based on the taxonomic ranking retrieved from the NCBI database (https://www.ncbi.nlm.nih.gov/taxonomy) for the bacterial **(A)** and fungal **(B)** species isolated from the Djoser pyramid (DP) and Lahun pyramid (LP). The abundance percentage of each order and class is demonstrated as a pie chart between both pyramids at each node. (Note: order Capnodiales node includes Mycosphaerellales and Cladosporiales species).

The ranking of bacterial and fungal species and the relationship of the organisms isolated from both pyramids was defined based on the taxonomic unrooted phylogenetic tree (genus level). In the bacterial species case, the class Actinobacteria ranked first in both pyramids, while it varied for Proteobacteria, which ranked third in DP and second in LP, and vice versa for Firmicutes. Species belonging to the genus *Clostridium* (order Clostridiales, class Firmicutes) were only found in DP; oppositely, *Agrobacterium* and *Rhizobium* species (order Rhizobiales, class Proteobacteria), *Micromonospora* sp. (order Micromonosporales, class Actinobacteria) and *Klebsiella* (order Enterobacteriales, class Proteobacteria) were only found in LP. Species of order Micrococcales (class Actinobacteria), species of genera *Bacillus* (order Bacillales, class Firmicutes), *Streptomyces* (order Streptomycetales, class Actinobacteria), and *Xanthomonas* (order Xanthomonadales, class Proteobacteria) were higher represented in DP than in LP. On the contrary, species from the genus *Pseudomonas* (order Pseudomonadales, class Proteobacteria) were higher in LP than in the DP (Figure 4A).

In the case of the fungal species, the class Dothideomycetes ranked; first, Sordariomycetes ranked second, followed by Eurotiomycetes in both pyramids. Species of genera *Glomerella* (order Glomerellales, class Sordariomycetes) and *Phialocephala* (order Ophiostomatales, class Sordariomycetes) were only found in DP. Oppositely, species of genera *Monilinia* (order Helotiales, class Leotiomycetes), *Mucor* (order Mucorales, class Mucoromycetes), and *Puccinia* (order Pucciniales, class Pucciniomycetes) were only found in LP. *Cladosporium* (order Cladosporiales), *Mycosphaerella* (order Mycosphaerellales), and *Pseudotaeniolina* (order Capnodiales) species, all belong to the class Dothideomycetes were represented in DP more than LP, and equally for *Alternaria*, *Curvularia*, and *Stemphylium* species (order Pleosporales, class Dothideomycetes), but slightly lower than Capnodiales. On the contrary, *Aspergillus* species (order Eurotiales, class Eurotiomycetes) were somewhat higher in LP than DP, and *Fusarium*, *Stachybotrys*, and *Trichoderma* species (order Hypocreales, class Sordariomycetes) were higher represented in LP than in DP (Figure 4B).

### 3.6. Culturable versus unculturable microbial species identification

**Figure 5:**
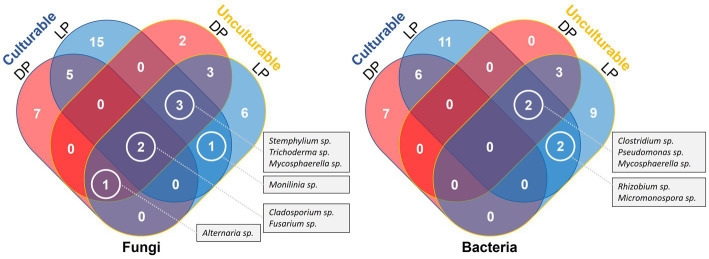
Venn Diagram illustrate a comparative listing of the culturable and culturable microbial species detected from both Djoser and Lahun Pyramids.

Except for few cases, most of the unculturable bacteria and fungi were of unknown species. When compared with the culturable species, no common species were detected by both methods, only unknown species identified at generic level. In case of bacteria, no taxa were detected by both methods from both pyramids, however, the culturable and unculturable methods yielded two unknown species belonging to *Rhizobium* and *Micromonospora* genera from Lahun Pyramid, and two additional unknown species belonging to *Clostridium* and *Pseudomonas* genera were found common with the unculturable species detected from Djoser Pyramid. In case of fungi, unknown species belonging to *Cladosporium* and *Fusarium* genera were detected by both methods from both pyramids. The culturable and unculturable methods yielded one unknown species belonging to genus *Monilinia* from Lahun Pyramid, and three additional unknown species belonging to *Stemphylium*, *Trichoderma*, and *Mycosphaerella* genera were found common with the unculturable species detected from Djoser pyramid. Unknown species belong to Alternaria were detected using culturable and unculturable methods from Djoser Pyramid and found common with the unculturable species found from Lahun Pyramid (Figure 5).

## 4. Discussion

There is no doubt that microorganisms greatly influence stone transformation and decay in a currently termed biodeterioration process. Unfortunately, such modification is irreversible, causing significant damage to human-made stone structures, such as monuments of cultural value and historical significance. Many reports concentrated on the diversity of the microbiota inhabiting such complex and harsh stone microhabitats (Urzì et al., 2000; Scheerer et al., 2009; Sanz et al., 2017; Pena-Poza et al., 2018). Those microorganisms are considered the second factor after erosion (i.e., physical and chemical damage), while other studies considered them the primary cause of decay and deterioration (Warscheid and Braams, 2000; Liu et al., 2020). Microorganisms inhabiting stone feature the extremotolerance aspect to survive and reproduce under such harsh conditions (Rampelotto, 2013).

The recorded microbial communities varied in composition, and the dominant species at bacterial and fungal levels between the two largest pyramids of the Memphis necropolis of ancient Egypt. However, the commonly recorded species were the key to defining the stone-inhabiting bacteria (SIB) and rock-inhabiting fungi (RIF). A total of 19 bacterial and 16 fungal species were exclusively culture-dependent, while 92 bacterial and 122 fungal species were culture-independent. The culture-dependent identification (traditional) method is considered less informative and convenient than the culture-independent identification, as the former enabled the detection of ≤5% of the total microbial community (Dakal and Arora, 2012).

Nevertheless, the traditional method has many benefits; the most important is the availability of the detected species and/or strains for further studies, especially when they are newly identified; however, the metabarcoding identification method has shown more advantages, considering that, in most cases, the behavior of a species is better explained in relation to the entire microbial community within a particular substrate (Li et al., 2016).

The concentration of eDNA extracted from arid and hyper-arid soils, as in the case described here, is very low (Schneegurt et al., 2003), which poses a challenge to the application of metabarcoding. In our study, bulking the samples increased the eDNA concentration and improved the 16S rRNA and ITS library preparation and sequencing. While the metabarcoding revealed higher microbial diversity than expected, considering the harsh arid conditions of the sampling sites (Lang-Yona et al., 2018). The most common bacterial class shared between the two pyramids is Actinobacteria, which is deemed to be the dominant bacteria in outdoor and subterranean habitats, such as caves and tombs (Cuezva et al., 2012). Concerning the highly represented families in both pyramids, the Planococcaceae, gram-positive bacteria with no known characteristics exclusive to all family members, dominate. However, the known isolated species was *P*, *salinarum*, which was previously isolated from a marine solar saltern and can grow *in vitro* up to 13% w/v NaCl (Yoon et al., 2010). The species representing the family Micrococcaceae was *Kocuria rosea*, known as a common soil and water species found previously in extreme environments such as heavily polluted waters, deep-sea sediments, and spacecraft surfaces (Coil et al., 2016). The most abundant species belonged to the family Geodermatophilaceae, described as one of the most abundant stones inhabiting Actinobacteria (Urzi et al., 2001; Sghaier et al., 2016). Two known species of the family Geodermatophilaceae, *B*, *aggregatus* and *B*, *saxobsidens*, that cause an orange coloration were identified from both pyramids. The latter was found to resist harsh environmental conditions (e.g., UV light, ionizing radiation, desiccation, and heavy metals (Gtari et al., 2012; Montero-Calasanz et al., 2015)).

Compared with the isolated culture-dependent bacterial species, the species of Geodermatophilaceae and Planococcaceae did not grow in the currently used media. Therefore, different media compositions and protocols will be required to retrieve such isolates, especially for the two unknown species found highly represented in both pyramids (*Blastococcus* sp. and *Planococcus* sp.). Chemoorganotrophic bacteria utilize a wide range of nutrients and may serve other microorganisms by breaking down poorly degradable compounds, which could otherwise not be utilized (Saier et al., 2008). One example is the species belonging to the genus *Bacillus*, four of which were isolated from both pyramids and frequently identified on stone buildings (Kiel and Gaylarde, 2006). An exclusive bacterium to LP belonged to the genus *Micromonospora*, which was occasionally reported from decayed stone (Ciferri et al., 2000), along with isolates from *Micropolyspora*, and *Streptomyces*, which were previously reported from a tomb in Tella Baste, Zagazig city in Egypt (Abdulla et al., 2008). Other isolates were best known as common environmental species of natural presence on soil [e.g., *Arthrobacter* (Eschbach et al., 2003)].

One of the most functional bacterial gene groups in the surveyed sites of both pyramids was the amino acid transport and metabolism; it could be very efficient in the survival in the studied sites as amino acid metabolism is associated with abiotic stress tolerance mechanisms in bacteria (Batista-Silva et al., 2019). One group is the Mycosporine-like amino acids, a family of intracellular compounds biosynthesized by the shikimic acid pathway to synthesize aromatic amino acids and are expressed under biotic and abiotic stresses [e.g., high UV exposure (Bhatia et al., 2011)]. Identifying related pathways would help to understand the stone-inhabiting mechanisms.

Based on ITS metabarcoding functional analysis, most of the detected fungi are naturally present in the soil. At the same time, some are molds, plant-pathogen species, and/or wood-inhabiting fungi (e.g., *Chaetomium globosum*). It is worth mentioning that the family Pleosporaceae (class Dothideomycetes) was one of the most abundant families in the studied pyramids, followed by the family Trichomeriaceae (class Eurotiomycetes), which was represented by *A*, *chlamydospora* and *A*, *oudemansii* species, that were previously reported from similar studies (Ruibal et al., 2009; Piñar et al., 2019), and an extremotolerant RIF, *K*, *karalitana* (Isola et al., 2016), respectively.

Compared with fungal culture-dependent isolation, seven common genera were observed at both pyramidal sites. The most remarkable isolated fungal species was a black meristematic fungus, *Pseudotaeniolina globosa* De Leo, Urzì & De Hoog, which was an extremotolerant ecotype adapted to harsh and arid conditions and was attributed to the family Teratosphaeriacea, order Capnodiales (Rizk et al., 2021). Meristematic growth is infrequent in the fungal kingdom and can be interpreted as a specific response to external stress (Isola et al., 2016). RIF usually are extremotolerant microorganisms that can tolerate abiotic stress such as drought, prolonged water deficiency, osmotic stress, extreme temperatures, UV radiation, and outer-space conditions (Sterflinger, 2006; Onofri et al., 2012; Zakharova et al., 2013; Sterflinger et al., 2014; Selbmann et al., 2015). RIFs are known for the dark-colour aspect and are very active agents causing noticeable alteration patterns and exfoliation of stone monuments with endolithic activity (Onofri et al., 2014; De Leo et al., 2019). Comparing our results with similar studies, several fungal genera detected in the Djoser and Lahun pyramids were previously reported from cultural heritage material. For example, *Alternaria*, *Aspergillus*, *Cladosporium*, *Epicoccum*, *Fusarium*, *Mucor*, *Penicillium*, and *Trichoderma* were reported, among others, from storage room objects in the Tianjin Museum, China (Liu et al., 2018; Soliman and Magdy, 2018), Etruscan tombs in Italy and ancient tombs of the Baekje Dynasty in the Republic of Korea (Caneva et al., 2020).

To know the real potential of biodeterioration caused by the isolated species, a follow-up study is currently in progress to characterize the stone substrate where the microorganisms found are inoculated and subjected to specific environmental conditions as well as the examination of possible biocidal treatments using organic and natural products without deteriorating side effects.

## 5. Conclusion

Our survey of two of the oldest and largest pyramids in Memphis necropolis of ancient Egypt, Djoser and Lahun pyramids, revealed that both pyramids are inhabited by potential biodeterioration agents, some known for their ability to transform the stone surface and rock formation, and potentially as dangerous as physical and chemical erosions. Our results confirm that the best methodological approach to identifying and studying a complex microbial community is by combining microscopy and molecular identification for culture-dependent microbes. Metabarcoding methods are ideal for culture-independent microbes by extracting DNA and/or RNA directly from the substratum and/or biomass. Based on both culturable and unculturable microbial identification methods, the identified SIB known to be related to biodeterioration were *B*, *aggregatus*, *B*, *saxobsidens*, and *Blastococcus* sp., while *B*, *alkalitelluris*, *B*, *persicus*, *P*, *salinarum*, and *Planococcus* sp. will need further investigation to examine their biodeterioration effect. Equally, the surveyed RIF in the current study were *K*, *karalitana* and *P*, *globosa*, in addition to a species belonging to the family Sporormiaceae, which will need further isolation and identification. These findings will require further inspection and biodeterioration-related analysis to fully understand the damaging effect on Djoser and Lahun pyramids and help to design prevention and conservation plans.

## Data availability statement

The datasets presented in this study can be found in online repositories. The names of the repository/repositories and accession number(s) can be found in the article/Supplementary material.

## Author contributions

MM, FL, OW, RR, and CU: conceptualization. SR and MM: data curation. MM: formal analysis. MM and MR: funding acquisition. SR and MM: investigation, methodology, and manuscript drafting. RR, OW, and CU: project administration. MM and RR: resources. OW, RR, FL, CU, and MR: revisions. All authors contributed to the article and approved the submitted version.

## Funding

SR was partially supported by a PhD exchange scholarship funded by the Erasmus Mundus Action 2: EMMAG Program (2014–2016), and the experiments were funded by Science, Technology and Innovation Funding Authority (STDF) under grant no. 26383 (2018–2020).

## Conflict of interest

The authors declare that the research was conducted in the absence of any commercial or financial relationships that could be construed as a potential conflict of interest.

## Publisher’s note

All claims expressed in this article are solely those of the authors and do not necessarily represent those of their affiliated organizations, or those of the publisher, the editors and the reviewers. Any product that may be evaluated in this article, or claim that may be made by its manufacturer, is not guaranteed or endorsed by the publisher.

## Footnotes

^1^ http://en.majorbio.com/

^2^ http://rdp.cme.msu.edu/

^3^ https://github.com/vegandevs/vegan

^4^ http://circos.ca/

^5^ https://orange.biolab.si/

^6^ https://www.ncbi.nlm.nih.gov/blast.cgi

^7^ https://www.ncbi.nlm.nih.gov/taxonomy
