# Supplementary material for: Culturable and unculturable potential heterotrophic microbiological threats to the oldest pyramids of the Memphis necropolis, Egypt
Source: Front Microbiol. 2023 May 18;14:1167083. doi: 10.3389/fmicb.2023.1167083 (PMC10232867; doi:10.3389/fmicb.2023.1167083)
Supplement: Supplementary file 1 [file Data_Sheet_1.docx]

**Supplementary files**

**Title:** Culturable and unculturable potential microbiological threat to the oldest pyramids of the Memphis necropolis, Egypt.

**Authors:** Samah Mohamed Rizk, Mahmoud Magdy, Filomena De Leo, Olaf Werner, Mohamed Abdel-Salam Rashed, Rosa M. Ros, Clara Urzì

**Supplementary Table 1.** Raw reads statistics obtained from Illumina run of the 16S rDNA.

| DNA Bulk Code | Sequence reads | Base number | Mean length | Min. length | Max. length |
| --- | --- | --- | --- | --- | --- |
| DP_S1 | 42,470 | 17,527,690 | 413 | 401 | 441 |
| DP_S2 | 41,166 | 17,077,794 | 415 | 339 | 439 |
| LP_S1 | 43,189 | 18,179,685 | 421 | 226 | 471 |
| LP_S2 | 48,697 | 20,536,504 | 422 | 261 | 526 |

**Supplementary Table 2.** Raw reads statistics obtained from Illumina run of the partial ITS region.

| DNA Bulk Code | Sequence reads | Base number | Mean length | Min. length | Max. length |
| --- | --- | --- | --- | --- | --- |
| DP_S1 | 54,512 | 13,662,045 | 251 | 166 | 331 |
| DP_S2 | 44,455 | 11,295,272 | 254 | 187 | 515 |
| LP_S1 | 71,434 | 17,006,153 | 238 | 147 | 343 |
| LP_S2 | 62,872 | 16,523,855 | 263 | 140 | 504 |

**Supplementary Table 3.** Molecular identification based 16S rDNA sequencing for the successful isolates and its percentages from Djoser (DP) and Lahun (LP) pyramids samples. Shaded rows are unique taxa isolated from one of the two pyramids.

| **Bacteria** | **Isolates %** | | | | | | | | | | | |
| --- | --- | --- | --- | --- | --- | --- | --- | --- | --- | --- | --- | --- |
|  | **Djoser Pyramid** | | | | | | **Lahun Pyramid** | | | | | |
|  | **DP1** | **DP2** | **DP3** | **DP4** | **DP5** | **DP6** | **LP1** | **LP2** | **LP3** | **LP4** | **LP5** | **LP6** |
| ***Agrobacterium tumefaciens*** (Smith and Townsend 1907) Conn 1942 | - | - | - | - | - | - | 25 | - | - | - | - | - |
| ***Arthrobacter agilis*** (Ali-Cohen 1889) Koch et al. 1995 | - | - | - | - | - | - | - | 30.8 | - | - | - | - |
| ***Arthrobacter parietis*** Heyrman et al. 2005 | - | - | - | 58.8 | - | - | - | - | - | - | - | 16.7 |
| ***Bacillus flexus*** (ex Batchelor 1919) Priest et al. 1989 | - | - | - | - | - | - | 12.5 | - | - | - | - | - |
| ***Bacillus pocheonensis*** Ten et al. 2007 | - | - | - | - | - | - | - | - | - | 16.7 | 30 | - |
| ***Bacillus safensis*** Satomi et al. 2006 | - | 19.5 | - | - | 40.5 | - | - | - | - | - | - | - |
| ***Bacillus subtilis*** (Ehrenberg 1835) Cohn 1872 | - | - | 35 | 26.5 | - | - | - | 7.7 | - | - | - | - |
| ***Brevibacterium iodinum*** (ex Davis 1939) Collins et al. 1981 | - | - | - | - | 10.8 | - | - | - | - | - | - | - |
| ***Brevibacterium*** Breed 1953 **sp.** | - | - | - | - | - | - | - | 34.6 | - | - | - | - |
| ***Clostridium*** Prazmowski 1880 **sp.** | - | - | - | - | - | - | - | 7.7 | - | - | - | - |
| ***Klebsiella oxytoca*** (Flugge 1886) Lautrop 1956 | - | - | - | - | - | - | 12.5 | - | - | 40 |  |  |
| ***Kocuria sediminis*** Bala et al. 2012 | 21 | - | 27.5 | - | - | - | - | 19.2 | 14 | - | - | 5.6 |
| ***Kocuria*** Stackebrandt et al. 1995 **sp.** | - | 12.2 | - | - | - | - | - | - | - | 30 | - | - |
| ***Kocuria turfanensis*** Zhou et al. 2008 emend. Camacho et al. 2017 | 52 | 29.3 | - | - | 48.9 | - |  |  |  |  |  |  |
| ***Micrococcus luteus*** (Schroeter 1872) Cohn 1872 | - | - | 17.5 | - | - | - | 22.5 | - | 20 | 6.7 | - | - |
| ***Micromonospora*** Orskov 1923 **sp.** |  |  |  |  |  |  | - | - | 22 | - | 35 | 22.2 |
| ***Pseudomonas fluorescens*** Migula 1895 |  |  |  |  |  |  | - | - | - | - | 15 | 11.1 |
| ***Pseudomonas oryzihabitans*** Kodama et al. 1985 | - | - | - | 14.7 | - | - |  |  |  |  |  |  |
| ***Pseudomonas*** Migula 1894 emend. Yang et al. 2013 **sp.** | - | - | - | - | - | - | - | - | 12 | - | - | - |
| ***Rhizobium*** Frank 1889 **sp.** | - | - | - | - | - | - | 20 | - | - | - | - | - |
| ***Streptomyces flavoviridis*** (ex Preobrazhenskaya et al.) Preobrazhenskaya 1986 | - | - | - | - | - | - | - | - | 12 | - | - | - |
| ***Streptomyces griseoflavus*** (Krainsky 1914) Waksman and Henrici 1948 | - | 22 | - | - | - | 53.8 | - | - | - | - | - | - |
| ***Streptomyces griseus*** (Krainsky 1914) Waksman & Henrici 1948 | - | - | - | - | - | - | - | - | 16 | - | - | - |
| ***Streptomyces heliomycini*** (ex Braznikova et al. 1958) Preobrazhenskaya 1986 | - | 17.1 | - | - | - | - | - | - | - | - | 20 | 11.1 |
| ***Streptomyces lavendulocolor*** (Kuchaeva et al. 1961) Pridham 1970 | - | - | - | - | - | - | - | - | - | - | - | 33.3 |
| ***Streptomyces marokkonensis*** Bouizgarne et al. 2009 | - | - | - | - | - | 46.2 | - | - | - | - | - | - |
| ***Xanthomonas campestris*** (Pammel 1895) Dowson 1939 | 27 | - | 20 | - | - | - | - | - | - | - | - | - |
| ***Xanthomonas*** Dowson 1939 **sp.** | - | - | - | - | - | - | - | - | - | 6.7 | - | - |
| **Un-identified** | - | - | - | - | - | - | 7.5 | - | 4 | - | - | - |

**Supplementary Table 4.** Molecular identification based on ITS2 region sequencing for the successful isolates and its percentages from Djoser (DP) and Lahun (LP) pyramids samples. Shaded rows are unique taxa isolated from one of the two pyramids.

| **Fungi** | **Isolate %** | | | | | | | | | | | |
| --- | --- | --- | --- | --- | --- | --- | --- | --- | --- | --- | --- | --- |
|  | **Djoser Pyramid** | | | | | | **Lahun Pyramid** | | | | | |
|  | **DP1** | **DP2** | **DP3** | **DP4** | **DP5** | **DP6** | **LP1** | **LP2** | **LP3** | **LP4** | **LP5** | **LP6** |
| ***Alternaria alternata* (Fr.) Keissl.** | - | - | - | - | - | - | 16.7 | - | - | - | - | - |
| ***Alternaria iridiaustralis*** E. G. Simmons, Alcorn & C. F. Hill | - | - | - | - | - | - | - | - | - | - | 6.7 | - |
| ***Alternaria obovoidea*** (E.G. Simmons) Woudenb. & Crous | - | - | - | 30 | 21.6 | 9.6 | - | 13.2 | 20 | - | - | - |
| ***Alternaria*** Nees **sp.** | - | - | - | - | - | 5.5 | - | - | - | - | - | - |
| ***Aspergillus flavus*** Link | - | - | - | - | - | 11 | - | - | - | - | - | - |
| ***Aspergillus niger*** Tiegh. | - | - | - | - | 9.8 | - | - | - | - | - | 6.7 | - |
| ***Aspergillus sydowii*** (Bainier & Sartory) Thom & Church | - | - | - | - | - | - | 20 | - | - | - | - | - |
| ***Aspergillus terreus*** Thom | - | - | - | - | - | - | - | 10.5 | - | - | 11.1 | - |
| ***Chaetomium globosum*** Kunze | - | - | - | - | - | - | 12.2 | - | - | 17.1 | - | 12.5 |
| ***Cladosporium halotolerans*** Zalar, de Hoog & Gunde-Cim. | - | - | - | - | - | - | - | - | - | - | 6.7 | 20 |
| ***Cladosporium* Link sp.** | - | - | 20 | 26.7 | 13.7 | 23.4 | 13.4 | - | - | - | - | - |
| ***Cladosporium velox*** Zalar, de Hoog & Gunde-Cim. | - | - | - | - | - | - | - | 23.7 | - | - | - | - |
| ***Cladosporium fulvum*** Cooke | - | - | - | - | - | - | - | - | - | 8.6 | - | - |
| ***Curvularia lunata*** (Wakker) Boedijn | - | - | - | - | - | 9.6 | - | - | 10 | 43.8 | - | - |
| ***Epicoccum nigrum*** Link | - | 83.3 | - | - | 7.8 | - | - | 15.8 | 16.7 | - | - | 27.5 |
| ***Epicoccum*** Link **sp.** | - | - | - | - | 10.4 | 5.5 | - | - | - | - | - | - |
| ***Fusarium equiseti*** (Corda) Sacc. | - | - | - | - | - | 8.2 | - | - | - | - | - | - |
| ***Fusarium oxysporum*** Schltdl. | - | - | - | - | - | - | 5 | - | - | - | - | 12.5 |
| ***Fusarium*** Link **sp.** | - | - | - | - | 13.7 | - | - | - | - | 10.5 | 8.9 | - |
| ***Glomerella cingulata*** (G. F. Atk.) Spauld. & H. Schrenk | - | - | - | - | - | 6.8 | - | - | - | - | - | - |
| ***Monilinia*** Honey **sp.** | - | - | - | - | - | - | - | 7.9 | - | - | - | - |
| ***Mucor ambiguous*** Vuill. | - | - | - | - | - | - | - | 5.3 | - | 14.3 | - | - |
| ***Mycosphaerella*** Johanson **sp.** | - | - | - | - | - | - | 7.77 | - | - | - | - | - |
| ***Mycosphaerella tassiana*** (De Not.) Johanson | - | - | - | - | - | - | - | - | 23.3 | - | 8.9 | - |
| ***Penicillium*** Link ***s*p.** | - | - | - | - | 13.4 | - | - | - | - | - | - | - |
| ***Phialocephala fluminis*** Shearer, J. L. Crane & M. A. Mill. | - | 16.6 | - | - | - | - | - | - | - | - | - | - |
| ***Podospora anserina*** (Rabenh.) Niessl | - | - | - | - | - | - | - | - | - | - | 20 | - |
| ***Pseudotaeniolina globosa*** De Leo, Urzì & De Hoog 2003 | - | - | 57.8 | - | - | 20.5 | - | - | - | - | - | - |
| ***Puccinia graminis*** Pers. | - | - | - | - | - | - | - | - | - | - | 2.2 | - |
| ***Stachybotrys chlorohalonatus*** B. Andersen & Thrane | - | - | - | - | - | - | - | - | 30 | - | 6.7 | 10 |
| ***Stemphylium*** Wallr. **sp.** | - | - | - | - | - | - | 10 | 18.4 | - | - | - | - |
| ***Trichoderma*** Pers. **sp.** | - | - | - | - | - | - | 6.7 | - | - | - | - | - |
| ***Ulocladium botrytis*** Preuss | - | - | - | - | - | - | - | - | - | - | 15.6 | - |
| ***Ulocladium*** Preuss **sp.** | - | - | 22.2 | 43.3 | 9.5 | - | - | - | - | 5.71 | - | - |
| **Un-identified** | - | - | - | - | - | - | 7.8 | 5.3 | - | - | 6.7 | 17.5 |


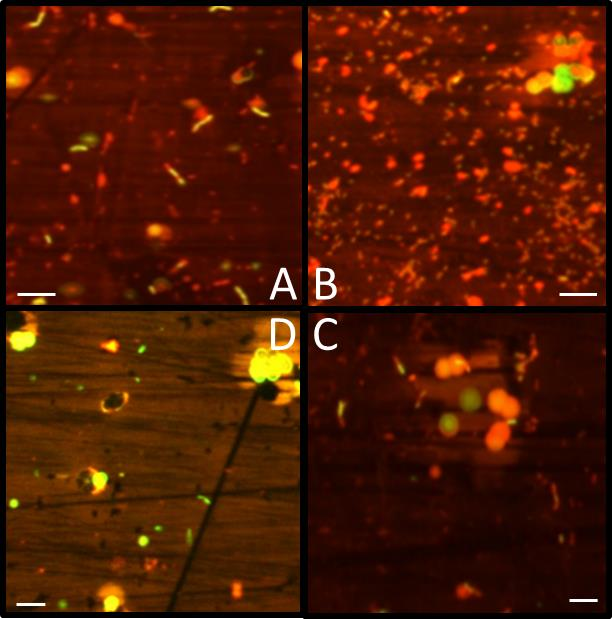


**Supplementary Figure 1.** Epifluorescent microscopic observation of some samples stained with Acridine Orange on a slide. Phototrophic cytoplasm shows a red autofluorescence; the nuclei of eukaryotic cells appeared green, while the cytoplasm of heterotrophs was orange. A: mixed microbial community mainly prokaryotic with some eukaryotic presence from DP2. B: from DP5 site showing mixed microbial community mainly composed of phototrophs with a denser eukaryotic presence of green color in the upper corner. C: sample from LP2 site showing the dominant presence of eukaryotic microorganisms with low phototrophs color signals. D: examined sample from LP4 site showing a mixed microbial community of bacteria in red clumps and fungal or algae cells in green (bar = 10 μm).


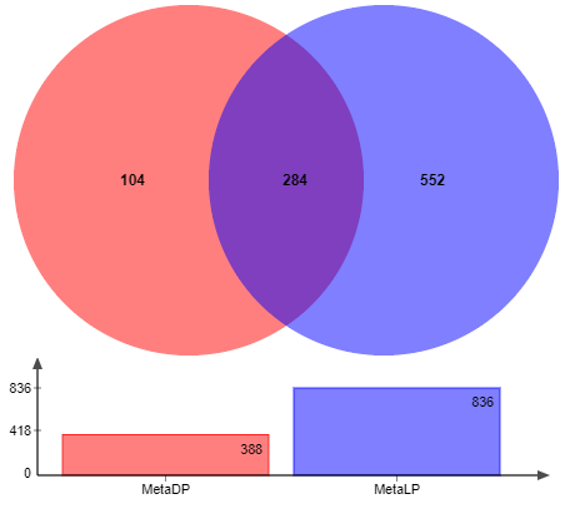


**Supplementary Figure 2.** Histogram and Venn diagram plot for the identified OTUs based on bacterial 16S rDNA metabarcoding analysis from the two pyramids DP (red) and LP (blue).


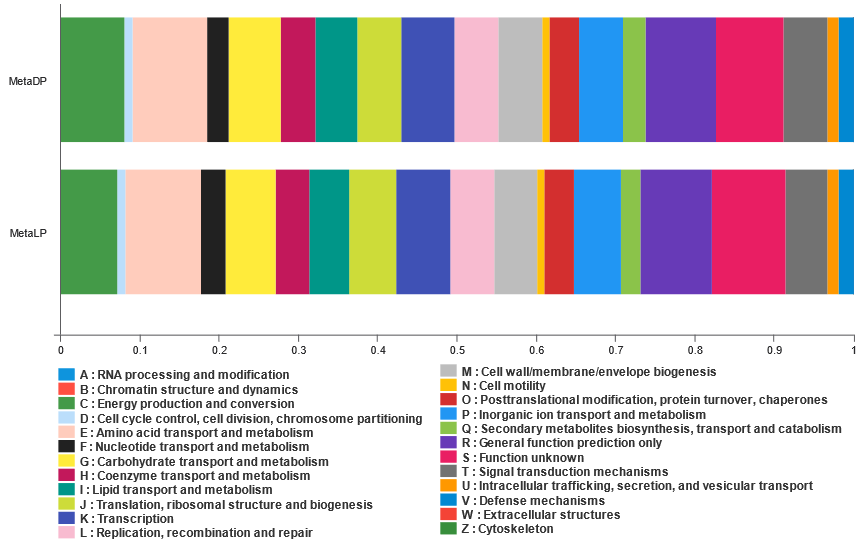


**Supplementary Figure 3.** Functional profile of the bacterial community of both pyramids, DP and LP, based on the identified OTUs in the metabarcoding samples. Each functional group is colored and lettered alphabetically from A to Z**.** The relative abundance of each function is indicated.


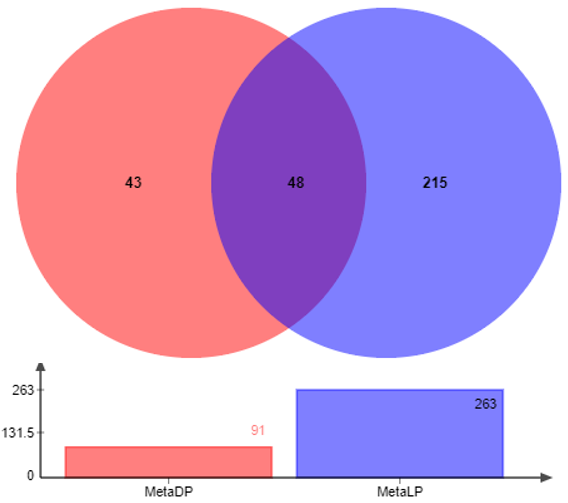


**Supplementary Figure 4.** Histogram and Venn diagram plot for the identified OTUs based on the ITS2 region metabarcoding analysis from the two pyramids DP (red) and LP (Blue).


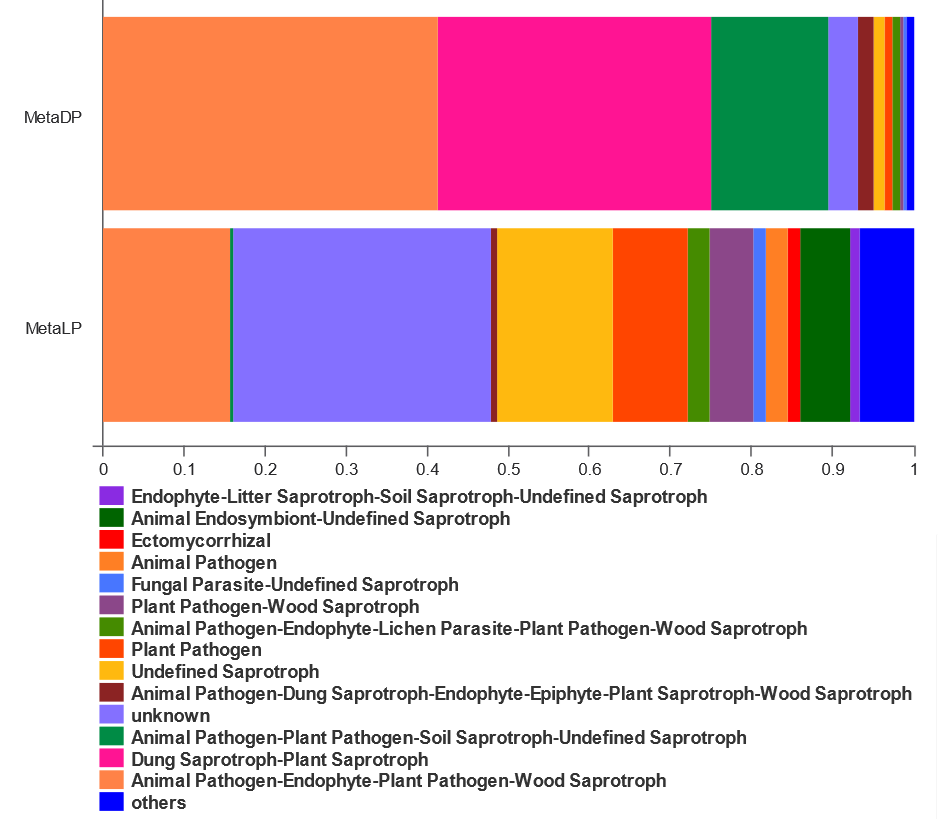


**Supplementary Figure 5.** Functional profile of the fungal community of both pyramids, DP and LP, based on the identified OTUs in the metabarcoding samples. Each functional group is colored. The relative abundance of each function is indicated.
